# Indigenous Lactic Acid Bacteria as Antioxidant Agents in the Production of Organic Raw Fermented Sausages

**DOI:** 10.3390/antiox13111305

**Published:** 2024-10-27

**Authors:** Anna Łepecka, Piotr Szymański, Anna Okoń

**Affiliations:** Department of Meat and Fat Technology, Prof. Wacław Dąbrowski Institute of Agricultural and Food Biotechnology—State Research Institute, 02-532 Warsaw, Poland; piotr.szymanski@ibprs.pl (P.S.); anna.okon@ibprs.pl (A.O.)

**Keywords:** LAB, meat products, *Lactobacillus*, *Lactiplantibacillus*, *Pediococcus*, antioxidative, properties, DPPH, ABTS, autochthonous

## Abstract

The study aimed to assess the impact of lactic acid bacteria (LAB) strains on the antioxidant, physico-chemical properties, and microbiological quality of fermented sausages. Five treatments of raw sausages were prepared: two controls without LAB addition (C, P), and three samples with LAB addition (SCH1, BAL6, KL14). Fatty acid composition, cholesterol content, physico-chemical, microbiological tests, and antioxidant assays, were performed at time 0 and after 1 and 2 months of storage. A significantly higher ability to scavenge free radicals of DPPH (2,2-diphenyl-1-picrylhydrazyl) was found in sausages with all LAB strains. In the case of the ABTS (2,2′-azino-bis(3-ethylbenzothiazoline-6-sulfonic acid)) test, it was noted that KL14 treatment had higher antioxidant activity. The main fatty acids in sausages were monounsaturated and saturated. A significantly lower cholesterol content was observed in sausages with the addition of LAB. Sausages with LAB strains differed significantly in pH value. Water activity decreased significantly during storage. After 2 months of storage, the sausages with BAL6 and KL14 strains were characterized by significantly lower redox potential and a lower TBARS (thiobarbituric acid reactive substances) index. It was found that P sausages had the darkest color. SCH1, BAL6, and KL14 strains were also capable of producing red color. The total number of microorganisms in the sausages was high, which is mainly due to the high LAB content and yeast and mold counts. No spoilage or pathogenic microflora were detected. Indigenous LAB strains have the potential to improve the quality and safety of fermented meat products.

## 1. Introduction

Human consumption of meat is currently controversial. On the one hand, it is an important element in the food economy, and on the other, it increases the intensification of animal breeding. This, in turn, increases the number of animals and also has a significant negative impact on the environment [1]. Excessive, uncontrolled meat consumption is a threat to sustainable development. Modern meat production is a huge burden on the natural environment [2]. Additionally, a question should be asked about the ethical aspect of eating meat. We face a huge challenge, primarily aimed at reducing the use of antibiotics and caring for animal welfare and the natural environment, including reducing greenhouse gas emissions [3]. One of the possibilities for more sustainable meat production is organic production. This production minimizes the use of additional substances [4].

With the increase in consumer awareness of food and human nutrition, the search for high-quality food has become a fact. In the case of meat and meat products, consumers more and more often declare that they could consume less of it, in favor of high-quality products, i.e., those that are characterized by a unique taste or natural composition. At the same time, they are willing to pay more for high-quality products. Organic and traditionally produced meat products are gaining popularity and are an alternative to mass-produced products, especially those produced with a large number of additives [4,5].

The use of microorganisms to extend the shelf life of meat products has been known and practiced for hundreds of years. Meat products were preserved by spontaneous fermentation by naturally occurring microorganisms [6]. Currently, starter cultures are used on an industrial scale, but also on an artisanal level. Meat starters usually contain selected cultures of lactic acid bacteria (LAB), coagulase-negative staphylococci (CNS), yeasts, filamentous fungi, or Actinobacteria. The purpose of starter cultures for the production of fermented meat products is primarily to standardize the product, as well as to shorten the maturation time. In addition, they play a role in acidifying the matrix, produce antimicrobial substances, inhibit the development of spoilage and pathogenic microorganisms, allow for the reduction in the use of nitrites and nitrates, inhibit the formation of biogenic amines and polycyclic aromatic hydrocarbons, prevent excessive drying of the product, and are responsible for creating the taste, smell, color, texture and nutritional value of products [7,8,9]. Starter cultures are most often inoculated in freeze-dried or frozen form, after prior mixing in water or brine. The low fermentation temperature (below 25 °C) allows the acquisition of a high-quality product with a long maturation and shelf life, without the risk of the development of undesirable microorganisms [8,10].

Antioxidants are substances that, in low concentrations, delay the oxidation of easily oxidizable biomolecules such as lipids and proteins, thereby improving the shelf life of products by protecting them from deterioration caused by oxidation [5]. Chemically synthesized antioxidants are not accepted by consumers [11,12]. An interesting direction of research is the use of microorganisms with unique properties and technological features isolated from naturally fermented food and the environment in meat processing [6,13,14,15]. In addition to the probiotic properties of some LAB strains, they are capable of a sensitive and rapid response to oxidative stress. They can promote the production of antioxidant enzymes whose role is to remove reactive oxygen species and mitigate oxidative damage [16,17]. Our model tests indicate that selected indigenous LAB isolated from organic ripening meat products exhibit antioxidant activity [18]. These specific biochemical properties of bacteria can be used to inhibit oxidation processes in meat products, which are particularly susceptible to these adverse changes. This study aimed to determine the influence of indigenous lactic acid bacteria strains on the antioxidant properties and selected quality parameters of raw fermented sausages.

## 2. Materials and Methods

### 2.1. Materials

#### 2.1.1. Lactic Acid Bacteria Strains

The research material consisted of three LAB strains isolated from organic fermented meat products—pork roast, gammon, and sausage [19]. The strains *Lactiplantibacillus plantarum* (*L. plantarum*) SCH1 (GenBank accession KX014848), *Pediococcus pentosaceus* (*P. pentosaceus*) BAL6 (GenBank accession KX021347), and *Pediococcus pentosaceus* (*P. pentosaceus*) KL14 (GenBank accession KX021364) were selected for the production of organic raw fermented sausages based on previously conducted screening antioxidant tests. The following tests were performed: ability to scavenge DPPH free radicals, activity of scavenging ABTS radicals, activity of superoxide dismutase, resistance to superoxide anion radicals, resistance to hydroxyl radicals, ability to produce catalase, and resistance to hydrogen peroxide [18]. LAB strains were stored frozen at −80 °C in MRS broth (Oxoid, Basingstoke, UK) with the addition of 20% glycerol (*v*/*v*; Chempur, Piekary Śląskie, Poland). For the activation, the cultures were incubated in fresh MRS broth at 37 °C for 24 h.

Fresh, 24 h LAB starter cultures were used for the production of sausages. The counts of bacteria were as follows: SCH1 strain: 9.15 ± 0.21 log CFU/mL; BAL6 strain: 8.93 ± 0.36 log CFU/mL; KL14 strain: 8.44 ± 0.51 log CFU/mL (bacterial count was plate-checked by plating on MRS agar, Oxoid, Basingstoke, UK according to ISO 15214:1998 [20]). The MRS broth was then centrifuged at 6000 min^−1^ (RCF 3341× *g*) for 10 min (MPW Med. Instruments, Warsaw, Poland), and the biomass was suspended in sterile 0.85% NaCl solution (Pol-Aura, Dywity, Poland).

#### 2.1.2. Raw Fermented Sausages

The raw material (15 kg for one treatment) consisted of lean and fatty meat from pork ham (50/50%). The ratio of meat to fat was used to obtain model fermented sausages with a high fat content, to best highlight the oxidative changes taking place in the stored product. Five treatments were prepared: C—control treatment, with 2% salt, 0.5% glucose (Pol-Aura, Dywity, Poland), and 100 mL 0.85% NaCl (without the addition of LAB strains); P—control treatment, with 2% nitrite curing mixture (99.5% NaCl, 0.5% NaNO_2_), 0.5% glucose and 100 mL 0.85% NaCl (without the addition of LAB strains); SCH1—research treatment with 2% salt, 0.5% glucose and 100 mL mixture of *L. plantarum* SCH1 biomass suspended in 0.85% NaCl; BAL6—research treatment with 2% salt, 0.5% glucose, and 100 mL mixture of *P. pentosaceus* BAL6 biomass suspended in 0.85% NaCl; KL14—research treatment with 2% salt, 0.5% glucose, and 100 mL mixture of *P. pentosaceus* KL14 biomass suspended in 0.85% NaCl.

**Figure 1:**
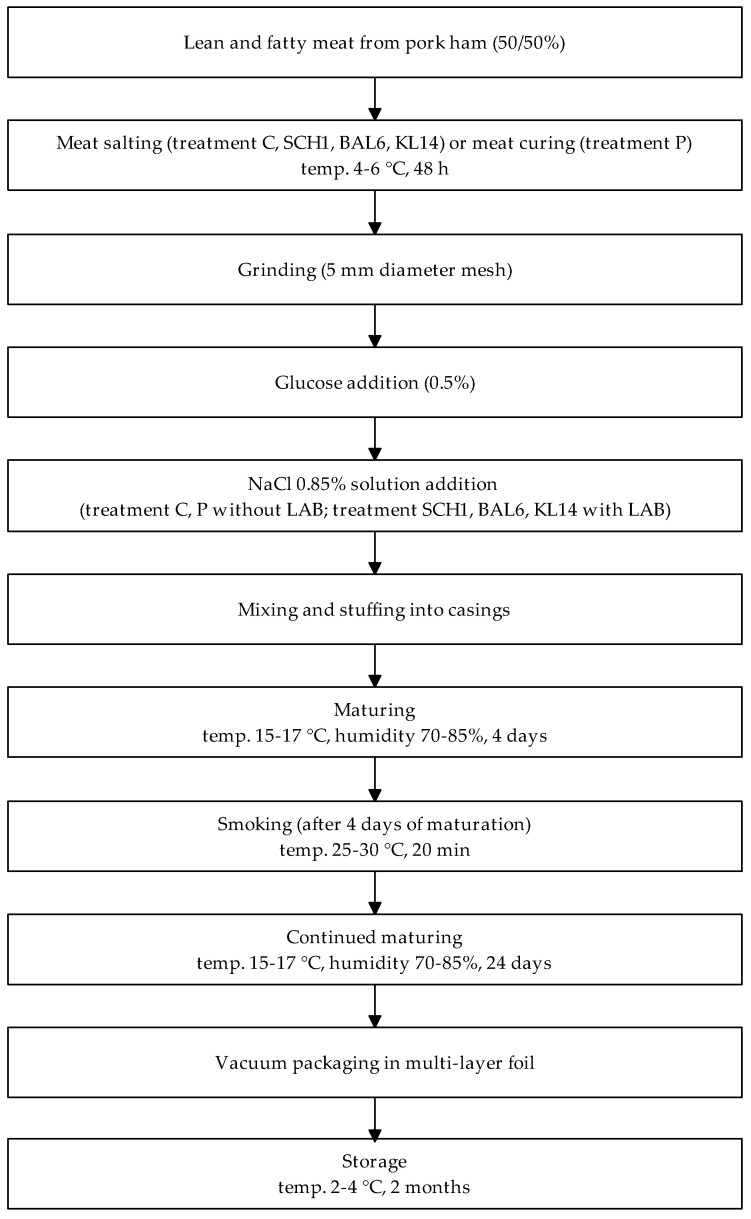
Technological process of producing raw fermented sausages.

In the first stage, the meat was salted (treatment C, SCH1, BAL6, KL14) or cured (treatment P) for 48 h in cooling conditions (4–6 °C). Then, the meat was ground in a grinder (5 mm diameter mesh), glucose and NaCl solution (with or without the addition of LAB) were added, and it was mixed and stuffed into casings. The sausages were matured at 15–17 °C and 70–85% humidity for 4 days. After that, the sausages were smoked (25–30 °C, 20 min) in a KWP2/G smoking and steaming chamber (REX-POL Sp. z o.o., Chorzów, Poland). Next, the maturing was continued for 24 days (15–17 °C, 70–85% humidity). The final meat products were vacuum-packed in a polyamide and polyethylene multilayer film. The sausages were refrigerated at 2–4 °C for 2 months. Figure 1 presents the technological process of producing raw fermented sausages.

Finally, in meat stuffing, the number of added LAB oscillated about 4 log CFU/g of the product (the result was given by mathematical calculations). The produced sausages contained, on average, 32.4% water, 29.6% protein, 30.3% fat, <0.5% carbohydrates, and 3.24% NaCl. The contents of water, protein, fat, and carbohydrates, as well as the salt content, were determined based on the ISO standards [21,22,23,24].

Three independent production batches were made, and each determination was performed in three independent replicates. The tests were carried out three times: after production (after the entire production process, after maturing and packaging of the products)—time 0, after 1 month (time 1), and after 2 months of storage (time 2), except for the fatty acid composition and cholesterol content, which were determined at time 0 and 2.

### 2.2. Methodology

#### 2.2.1. Total Antioxidant Capacity (TAC)

The hydrophilic fraction was used to determine the Total Antioxidant Capacity (DPPH assay, ABTS assay).

##### Extraction of the Hydrophilic Fraction

Hydrophilic fractions were prepared according to the methodology of Sacchetti et al. [25]. Samples of sausages (50 g) were added to 100 mL of distilled water and homogenized using a T18 digital ULTRA-TURRAX^®^ homogenizer (IKA Poland Sp. z o.o., Warsaw, Poland) in an ice bath. The homogenate was placed in a 100 mL vial wrapped in aluminum foil and extracted in a shaker at 4 °C for 1 h. Then, the meat homogenate was centrifuged at 3300 min^−1^ for 35 min (MPW Med. Instruments, Warsaw, Poland). The supernatant was decanted into new tubes.

##### Preparation of the Antioxidant Standard

Trolox (6-hydroxy-2,5,7,8-tetramethylchroman-2-carboxylic acid; Merck KGaA, Darmstadt, Germany) was used as an antioxidant standard for ABTS (2,2′-azino-bis(3-ethylbenzothiazoline-6-sulfonic acid). Standard solutions of Trolox (0.0; 0.2; 0.4; 0.6; 0.8; 1.0; 1.2 mM) in 5 mM PBS (Phosphate Buffered Saline; pH 7.4; 30 °C; Merck KGaA, Darmstadt, Germany) were prepared [26].

##### DPPH Assay

The radical scavenging activity of DPPH (2,2-diphenyl-1-picrylhydrazyl; 0.2 mM; Merck KGaA, Darmstadt, Germany) was evaluated for the aqueous homogenate obtained from meat according to the method of Blois [27], with modifications by Jang et al. [28]. Subsequently, 200 μL of the homogenate was added to 800 μL of water and 1 mL of methanolic DPPH. The mixture was vortexed and left at room temperature for 30 min. A tube containing 1 mL of distilled water and 1 mL of DPPH (0.2 mM) served as a control. The absorbance was measured at 517 nm (Hitachi U-2900, Tokyo, Japan). The radical scavenging of DPPH is given in %.

DPPH radical scavenging activity (%) = [1 − (A/A_1_)] × 100% (1)

where A—absorbance value of the tested solution, A_1_—absorbance value of the control solution.

##### ABTS Assay

Formation of the ABTS^+^ radical cation was initiated by reacting 14 mM ABTS (Merck KGaA, Darmstadt, Germany) with an equal volume of 4.9 mM potassium persulfate (Merck KGaA, Darmstadt, Germany). The solution was incubated in the dark at room temperature for 12–16 h. Before analysis, the absorbance of the ABTS^+^ solution was measured at 734 nm and adjusted to 0.700 (±0.02) using 5.5 mM PBS (pH 7.4, temp. 30 °C). A 10 µL sample of meat homogenate or Trolox standard (0.0–1.2 mM in PBS) was added to 1.0 mL of ABTS^+^ solution and mixed thoroughly, and the absorbance at 734 nm was read after 1 min (Hitachi U-2900, Tokyo, Japan). Then, the second measurements were taken after 6 min of incubation at 30 °C. The percent inhibition of blank absorbance was calculated for the Trolox standard and the meat sample, respectively [26,29]. The result is expressed as mM Trolox equivalent/g of product.

#### 2.2.2. Composition of Fatty Acids

Fatty acids were determined by gas chromatography using HP/Agilent 6890 II with a flame ionization detector (FID; Hewlett-Packard; Palo Alto, CA, USA) according to ISO 12966-1:2014 [30] and Okoń et al. [31]. Results are expressed as total saturated fatty acids (SFA), total monounsaturated fatty acids (MUFA), total polyunsaturated fatty acids (PUFA), total trans fatty acids (trans), total n-3 fatty acids (n-3), and total n-6 fatty acids (n-6). The result is presented in %.

#### 2.2.3. Cholesterol

The cholesterol content was determined by extraction of the lipid fraction, esterification of fatty acids, and derivatization of cholesterol in the presence of an internal standard. The sample was analyzed by gas chromatography with flame ionization detection (GC-FID). The result is presented in mg/100 g of product.

#### 2.2.4. pH Value

pH was determined according to ISO 2917:1999 [32] using a FiveEasy F20 pH meter with an LE438 electrode (Mettler-Toledo GmbH, Greifensee, Switzerland). A 10 g sample was homogenized (Bamix SwissLine M200; Bamix Polska, Sławno, Poland) with 50 mL of distilled water. Measurements were carried out at 20 °C.

#### 2.2.5. Water Activity

Water activity was determined according to ISO 18787:2017 [33] using the Aqualab Pawkit DE201 apparatus (METER Group, Inc., Pullman, WA, USA). Measurements were carried out at 20 °C.

#### 2.2.6. Oxidation-Reduction Potential (ORP)

ORP was determined using a SevenCompactTM S220 device with an InLab Redox electrode (Mettler-Toledo GmbH, Greifensee, Switzerland). A 10 g sample was homogenized (Bamix SwissLine M200; Bamix Polska, Sławno, Poland) with 50 mL of distilled water [31]. The obtained results were converted to the ORP value with the standard hydrogen electrode Eh (mV). Measurements were carried out at 20 °C. The result is expressed in mV.

#### 2.2.7. TBARS (Thiobarbituric Acid Reactive Substances) Index

The fat oxidation TBARS index was determined by measuring the absorbance value of the solution of meat product and 2-thiobarbituric acid, as described by Pikul et al. [34]. The color intensity of the reaction of malondialdehyde (MDA) and 2-thiobarbituric acid was measured using a U-2900 spectrophotometer (Hitachi, Tokyo, Japan) at a wavelength of 532 nm. The TBARS value is expressed in mg MDA/kg of product.

#### 2.2.8. Color Measurement

The color was measured in the CIE L*a*b* system. Instrumental color measurement was performed using a Minolta CR-300 reflectance spectrophotometer (Konica Minolta, Tokyo, Japan) and the parameters are described according to Tomasevic et al. [35]. The aperture size was 8 mm, the observation angle was 2°, and the D65 illuminator and a pulsed xenon lamp were used as the default light source. Calibration was performed with a white standard (L* 99.18, a* −0.07, b* −0.05). Sausages were sliced into 12 mm thick pieces and twenty measurements were made for each sample.

#### 2.2.9. Microbial Evaluation

Microbiological analysis was performed using the plate method. Total viable count (TVC) was determined on nutrient agar (Oxoid, Basingstoke, UK) according to ISO 4833-1:2013 [36]. Lactic acid bacteria (LAB) count was determined on MRS agar (Oxoid, Basingstoke, UK) according to ISO 15214:1998 [20]. *Enterobacteriaceae* (ENT) count was determined on MacConkey agar (Oxoid, Basingstoke, UK) according to ISO 21528-2: 2017 [37]. *Escherichia coli* (EC) count was determined on TBX agar (Oxoid, Basingstoke, UK) according to ISO 16649-1:2018 [38]. The number of coagulase-positive staphylococci (*Staphylococcus aureus* and other species) (ST) was determined on Baird-Parker egg yolk tellurite agar (Oxoid, Basingstoke, UK) according to ISO 6888-1:2021 [39]. The number of yeasts and molds (YM) was determined on YGC agar (Yeast Glucose Chloramphenicol, Merck KGaA, Darmstadt, Germany) according to ISO 21527-2:2008 [40].

The presence of *Salmonella* spp. (SAL) was determined on XLD agar (Oxoid, Basingstoke, UK) according to ISO 6579-1:2017/Amd.1:2020 [41]. The presence of *Campylobacter* spp. (CAMP) on Chromogenic *Campylobacter* LAB-AGAR™ agar (Biomaxima, Lublin, Poland) was determined following ISO 10272-1:2017 [42].

#### 2.2.10. Statistical Analysis

One-way analysis of variance (ANOVA) between individual treatments (5) and between storage times (3) was used for statistical studies. The results were compiled as mean and standard deviation (±SD). The probability level of *p* < 0.05 was used to test the statistical significance of all experimental data. Tukey’s post hoc HSD test was used to determine the significance of mean values for multiple comparisons. Cluster analysis was carried out using Ward’s agglomeration method. Statistica 13.1 (TIBCO Software Inc., Palo Alto, CA, USA) was used to perform the calculations.

## 3. Results

**Figure 2:**
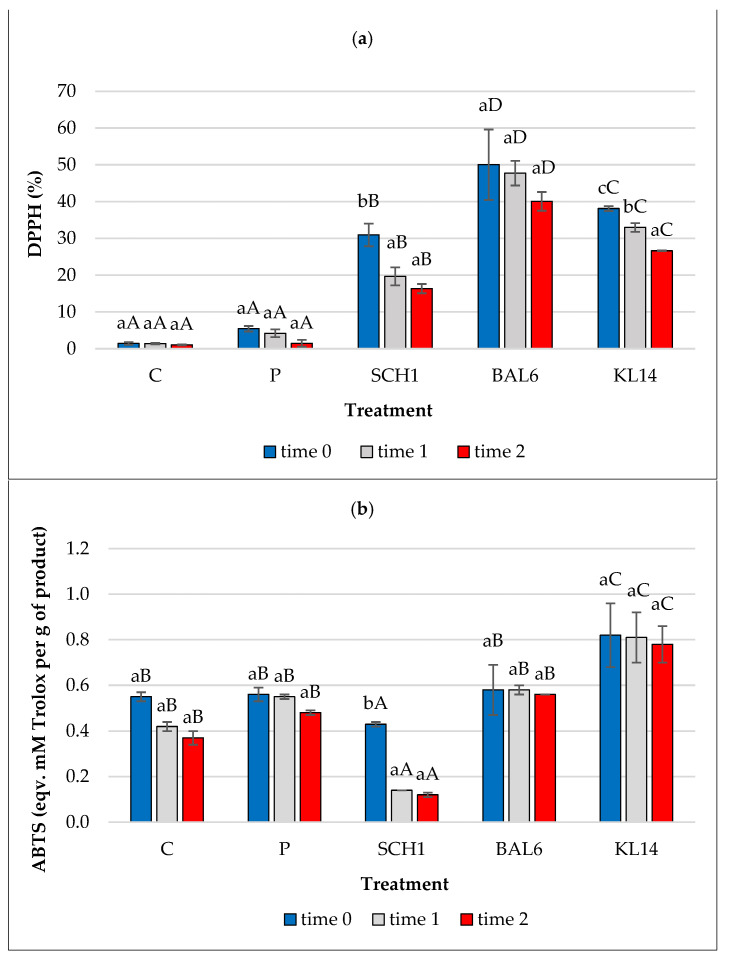
Total Antioxidant Capacity: (**a**) DPPH assay and (**b**) ABTS assay of the tested sausages. C—control treatment with salt, P—control treatment with salt nitrite, SCH1—treatment with *L. plantarum* SCH1; BAL6—treatment with *P. pentosaceus* BAL6; KL14—treatment with *P. pentosaceus* KL14. The values are expressed as means ± standard deviation; a–c statistically significant differences between times (*p* < 0.05); A–D statistically significant differences between treatments (*p* < 0.05).

Raw fermented sausages are meat products made in natural or artificial casings, from shredded meat and fat raw materials, cured or salted, with or without the addition of spices and starter cultures, smoked or unsmoked, raw, or subjected to a production maturing process [43]. Figure 2 shows the total antioxidant activity of the tested sausages. A significantly higher ability to scavenge free radicals of DPPH was found in the case of sausages with the addition of LAB strains (*p* < 0.05). After storage, a gradual decrease in this ability was observed, but only in the case of the KL14 treatment; this decrease was statistically significant (*p* < 0.05). A significantly higher ability to scavenge free radicals ABTS^+^ was shown by sausages with the addition of the KL14 strain (*p* < 0.05). A gradual decrease in ABTS was observed during 2-month storage. A significant reduction was observed for the SCH1 treatment (*p* < 0.05). The study of Karwowska et al. [44] also observed a decrease in total antioxidant activity during storage. The research of Wójciak et al. [45] found that specific protein breakdown products are involved in combating free radicals in meat. Meat and meat protein, in particular, is a good source of highly available amino acids. The antioxidant activity of the peptides is related to their metal ion chelation, lipid peroxidation inhibition, and radical scavenging properties [46,47,48]. In the presented research results, different values of antioxidant activity can be observed. Referring to the publication by Floegel et al. [49], in the case of food analysis, the results of the antioxidant capacity measurement may vary depending on the test used. Moreover, the authors indicate that the ABTS^+^ test better reflects the antioxidant content in various food products than the DPPH test. The ABTS^+^ test is based on the generation of blue-green ABTS^+^, which applies to hydrophilic and lipophilic systems. The DPPH test uses a radical dissolved in an organic environment and is therefore applicable to hydrophobic systems [49,50]. There is often no correlation between results obtained for the same material using different methods or even between results obtained for the same material using the same method in different laboratories. Chemical tests are performed in a non-physiological pH and temperature system. The bioavailability, dose, and metabolic transformation of the antioxidants in the living cell are not taken into account. This means that these methods may be subject to a large error [51]. We believe that the tests conducted using the ABTS^+^ method are closer to the antioxidant activity.

The tested sausages had a varied composition of fatty acids (Table 1). The highest content of monounsaturated acids (MUFA, 45.50–49.20%), followed by saturated (SFA, 36.40–40.85%) and polyunsaturated (PUFA, 10.15–17.60%) acids was found. The P treatment had a significantly different fatty acid content (*p* < 0.05). The differences were found in the SFA content (lower content) and PUFA content (higher content) compared to the other treatments (*p* < 0.05). The curing salt added to P treatment significantly affects lipid oxidation and the formation of volatile compounds. In the studies of Wang et al. [52], the curing salt promoted the formation of aldehydes from fatty acids. Free fatty acid (FFA) levels increase significantly during both drying and storage. The addition of nitrite and nitrate also affected lipid fractions, as a higher FFA content was found, resulting in a greater release of PUFA and MUFA [53]. There were no significant differences in the content of trans fatty acids, and their amount was small. No significant differences were noted in the content of n-3 acids (0.60–0.90%). A significantly higher content of n-6 acids was found in the case of the P and KL14 treatments (*p* < 0.05). Lipids are hydrolyzed during the fermentation process, creating free fatty acids, which are further oxidized to alcohols, ketones, aldehydes, esters, and others [54,55]. PUFA considered beneficial to human health are susceptible to oxidation, which can impair the taste and nutritional value of meat products. Moreover, in stored food, the main process responsible for quality degradation is lipid oxidation [56]. Due to the action of lipolytic enzymes on triacylglycerols and phospholipids, the amount of free fatty acids increases, and the higher level of PUFA results from the preferential hydrolysis of the outer fatty layer [57].

The significantly highest cholesterol content was found in the P treatment, and the significantly lowest cholesterol content was found in the KL14 treatment (*p* < 0.05). SCH1, BAL6, and KL14 strains can reduce the cholesterol content in sausages ripening during storage. One of the possible mechanisms of cholesterol reduction demonstrated by LAB is the deconjugation of bile salts. They may act by incorporating cholesterol into cell membranes, binding cholesterol on their cell surfaces, and assimilating cholesterol during growth [58,59].

Table 2 shows the pH value, redox potential, and TBARS index. Immediately after the production of sausages with the addition of SCH1, BAL6, and KL14 strains, they differed in pH value (5.50–5.56) compared to control C and P samples (5.41 and 5.65, respectively). Then, after 1 month of storage, a higher pH value (5.62–5.88) was found compared to time 0. The reason for this phenomenon may be the protein being broken down by proteinases, and therefore the formation of peptides, amino acids, and amines that neutralize organic acids [60]. After 2 months of storage, the pH value decreased again (5.50–5.85), which was statistically significant in the case of samples with LAB strains. Lowering the pH value during storage may be due to the influence of microorganisms that have the right conditions to produce lactic acid and other acids [61]. Similar observations were noted in the case of oxidation-reduction potential, where ORP decreased after 1 month of storage, and then increased after 2 months (*p* < 0.05). It was found that after 2 months of storage, the sausages with the addition of the BAL6 and KL14 strains were characterized by significantly lower ORP values (367.80 and 377.87 mV, respectively) than the control sausages (386.30–392.30 mV), which proves the oxidative stability of the sausages. The TBARS index is a sensitive and appropriate indicator to assess the formation of secondary products (malondialdehyde, aldehydes, ketones) and to assess the degree of lipid oxidation [62]. Substances reactive with thiobarbituric acid are formed as a by-product of lipid peroxidation (i.e., as fat degradation products). The TBARS values of the tested sausages were not high and were below 2 mg MDA/kg of product [63]. Initially, the C, P, BAL6, and KL14 sausages had a similar value of the index (0.41–0.48 mg MDA/kg of product), while the value of the index was significantly different for SCH1 treatment (1.53 mg MDA/kg of product). After 1 month of storage, the TBARS index decreased (*p* > 0.05), while after 2 months of storage, the control sausages (C and P) had a higher TBARS index, while the BAL6 and KL14 treatments had a lower one (0.36–0.39 mg MDA/kg of product). Similar research results were obtained in the studies of Chen et al. [62], where Harbin-dried sausages with the addition of LAB strains showed a lower TBARS index compared to control sausages. In turn, the research of Chmiel et al. [64] showed that the cooling of meat products has a positive effect on delaying lipid and protein oxidation processes during storage, which is an important clue to the storage parameters of this type of sausage.

The main reason for the deterioration of the quality of meat and meat products is lipid peroxidation. Salt can have a negative effect on meat products because it has a pro-oxidant effect. The mechanism of this process is not well understood, but a possible explanation is that sodium chloride may interfere with the structural integrity of the membrane to allow the catalysts easy access to the lipid substrates [57,65,66,67]. Curing is a meat preservation method widely used to extend the shelf life of meat products because nitrites delay lipid oxidation processes [57], while also having a significant impact on the formation of flavor compounds [68]. In the tested treatments of sausages with the addition of curing salt, a lower oxidation-reduction potential was found at time 0 and after 1 month of storage. After 2 months of storage, sausages with the addition of LAB strains were characterized by the lowest ORP. In turn, secondary oxidation products (TBARS) formed after 2 months of storage, which reached higher values in the case of P treatment than in C treatment. According to the literature, samples with the highest content of sodium nitrite should have the lowest TBARS value, which was not observed in our research. The main mechanism of the antioxidant action of nitrites is related to nitric oxide, which can trigger lipid oxidation by chelation of free radicals [44]. In the study of Trabelsi et al. [15], lower TBARS values in stored beef with the addition of probiotic *L. plantarum* strains (1.50–1.83 mg MDA/kg of meat) compared to control meat (2.26 mg MDA/kg of meat) were described. At the same time, a significant (*p* < 0.05) increase in the TBARS index was observed during refrigerated storage, which was also found in our research. According to Campo et al. [63], the threshold point for detecting a rancid taste and odor by the sensory panel is 2.00 mg MDA/kg of product. Referring to this research, after 2 months of storage, the TBARS index in SCH1 sausages exceeded the level of sensory acceptance (2.46 mg MDA/kg of product).

Table 3 presents the results of color measurement in the CIE L*a*b* system. As a result of the tests, it was found that the P treatment was characterized by the darkest color (parameter L* 42.82–46.64) among the tested sausages (*p* < 0.05). Interestingly, P treatment, involving the addition of curing salt, which has a significant effect on the development of red color in meat products [69], was characterized by a variable a* parameter. After a month of storage, the a* parameter decreased (redness decreased, *p* < 0.05), and after 2 months of storage the a* parameter increased (redness increased, *p* < 0.05). The characteristic discoloration of the red color of fermented sausages may be caused by oxidation. It is characterized by the conversion of MbFe^II^NO to nitrate and a brown derivative of metmyoglobin (MbFe^III^), and this process is associated with subsequent lipid oxidation and changes in redox potential [70,71]. Significant lightening of BAL6 and KL14 treatment was observed after 2 months of storage (*p* < 0.05). In general, sausages with the addition of LAB strains (L* 49.95–55.59) were lighter in color than control C and P sausages (L* 42.82–49.15). Similar research results were obtained by Hu et al. [14], where dried fermented sausages were characterized by a higher L* parameter for products with the addition of *Lactobacillus curvatus*, *L. plantarum*, and *Weissella hellenica* (*p* < 0.05). It was observed that in the case of KL14 treatment, the addition of LAB allowed the production of a red color in sausages, which was statistically similar to the color of cured products (P treatment). Lactic acid bacteria can create a red color in the product due to the interaction of nitric oxide with myoglobin; this creates nitrosomyoglobin, which is responsible for the characteristic red color of meat. LAB enzymes can stabilize the color of meat products [71]. In the case of SCH1 treatment, a significant increase in the b* parameter, which is responsible for the yellow color, was observed (*p* < 0.05). Browning of SCH1 sausages could also be observed visually.

The total number of microorganisms in the tested treatments of sausages was high and amounted to over 9 log CFU/g (Table 4). After 1 and 2 months of storage, there was a significant increase in total viable cells (TVCs) to over 10 log CFU/g (*p* < 0.05). The high TVC value is mainly due to the high number of lactic acid bacteria (LAB), whose number immediately after production was over 6.00 log CFU/g, and yeasts and molds (2.38–5.66 log CFU/g). It is a typical microbiota of fermented meat products [60,72,73,74]. After 1 month of storage, there was an increase in LAB for BAL6 and KL14 sausages (9.29 and 8.26 log CFU/g), and a decrease in LAB for C, P, and SCH1 sausages (6.15–6.22 log CFU/g). After 2 months of storage, a significantly higher LAB was found for sausages with the addition of BAL6 and KL14 strains (7.31 and 7.70 log CFU/g; *p* < 0.05). It is worth noting that in all the tested treatments of sausages, the number of lactic acid bacteria was high and amounted to over 6.00 log CFU/g throughout the entire refrigerated storage period. No *Enterobacteriaceae*, *Escherichia coli*, or *Staphylococcus aureus* (<1.00 log CFU/g) bacteria were found in the tested sausage treatments, and no presence of the bacteria *Salmonella* spp. and *Campylobacter* spp. was found. All raw fermented sausages were characterized by very good microbiological quality.

According to the literature, in traditional fermented meat products, fermentation occurs spontaneously and the developing microbiota is much more diverse, which depends on the quality of raw meat and the processing conditions used, such as curing salt, fermentation temperature, degree of acidification, and drying intensity [74]. The biochemical changes that occur during meat fermentation are aimed at improving the stability, quality, safety, and sensory characteristics of fermented products [7].

**Figure 3:**
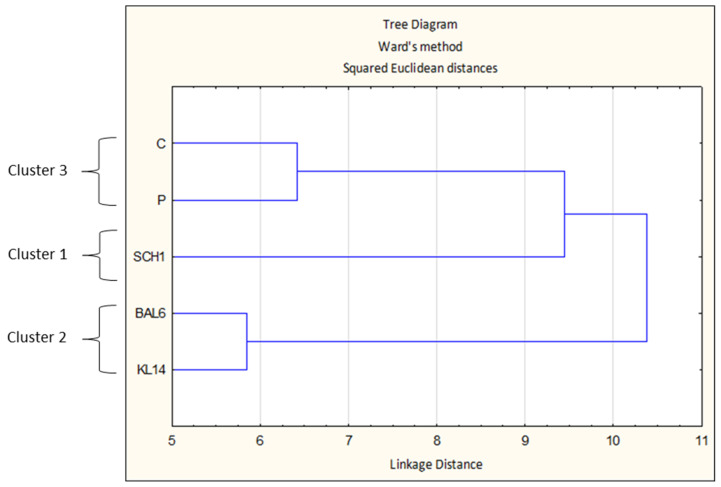
Cluster analysis using Ward’s agglomeration method. C—control treatment with salt, P—control treatment with salt nitrite, SCH1—treatment with *L. plantarum* SCH1; BAL6—treatment with *P. pentosaceus* BAL6; KL14—treatment with *P. pentosaceus* KL14; 1–3—clusters into which all tested sausages were grouped.

Cluster analysis using Ward’s agglomeration method distinguished three clusters, as shown in Figure 3. Cluster 1 contained sausages with the addition of the SCH1 strain. Cluster 2 contained sausages with the addition of BAL6 and KL14 strains, which at the same time belonged to the same bacterial species—*Pediococcus pentosaceus*. Cluster 3 contained sausages from control treatments C and P. It was found that sausages with the addition of LAB strains were not similar to control sausages, which indicates different technological properties of these sausages.

## 4. Conclusions

Fermentation is one of the oldest strategies to improve the safety and shelf life of products. Starter cultures are mainly represented by LAB, which can also be bioprotective factors controlling the fermentation process, native microbiota, and pathogen growth. Indigenous LAB strains have the potential for future use to improve the safety of fermented meats, even under conditions where chemical preservatives are limited or eliminated. In addition, research on environmental cultures is crucial to ensure the specific characteristics of traditional products that constitute an important cultural heritage.

This study demonstrated that the use of indigenous cultures of LAB with antioxidant properties has a beneficial effect on the quality characteristics of raw-ripened sausages. A significantly higher ability to scavenge DPPH free radicals was found in the case of sausages with the addition of LAB strains. In the case of the ABTS test, it was noted that KL14 treatment had higher antioxidant activity (*p* < 0.05). It was found that after 2 months of storage, sausages with the addition of BAL6 and KL14 strains were characterized by a significantly lower value of redox potential, which proves their oxidative stability. The TBARS value was not high, and after 2 months of storage, BAL6 and KL14 sausages were characterized by a lower TBARS index compared to the control treatments. The sausages produced with the addition of lactic fermentation bacteria were lighter in color, retaining the appropriate red color, and were characterized by appropriate microbiological quality, guaranteeing a long storage time.

To sum up, the use of indigenous lactic acid bacteria strains (*Pediococcus pentosaceus* BAL6, *Pediococcus pentosaceus* KL14) enables meat products with higher antioxidant potential, appropriate microbiological quality, and storage stability. LAB strains can be considered natural antioxidants, thanks to mechanisms such as regulation of redox potential, production of antioxidant metabolites, production of antioxidant enzymes, and ability to scavenge free radicals or chelating metal ions. Some of these properties were confirmed in the present study.

## Figures and Tables

**Table 1:** Composition of fatty acids and cholesterol content in the tested sausages.

Parameter	Time (Month)	Treatment				
		C	P	SCH1	BAL6	KL14
SFA (%)	0	40.85 ± 0.35 ^bB^	36.90 ± 0.28 ^bA^	40.40 ± 0.00 ^bB^	40.45 ± 0.07 ^bB^	39.10 ± 0.57 ^aB^
	2	40.45 ± 0.21 ^aC^	36.40 ± 0.14 ^aA^	40.25 ± 0.07 ^aC^	40.30 ± 0.00 ^aC^	39.05 ± 0.07 ^aB^
MUFA (%)	0	48.60 ± 0.42 ^aB^	45.50 ± 0.00 ^aA^	48.45 ± 0.07 ^aB^	49.15 ± 0.21 ^aB^	48.55 ± 0.07 ^aB^
	2	48.65 ± 0.14 ^aA^	45.60 ± 0.14 ^aA^	48.95 ± 0.21 ^bA^	49.20 ± 0.00 ^aB^	49.20 ± 0.14 ^bB^
PUFA (%)	0	10.40 ± 0.14 ^aA^	17.10 ± 0.14 ^aD^	10.85 ± 0.07 ^aB^	10.15 ± 0.21 ^aA^	12.05 ± 0.64 ^aC^
	2	10.80 ± 0.14 ^bB^	17.60 ± 0.14 ^bD^	10.90 ± 0.14 ^aB^	10.40 ± 0.00 ^bA^	12.50 ± 0.00 ^bC^
Trans (%)	0	0.20 ± 0.00 ^aA^	0.10 ± 0.00 ^aA^	0.20 ± 0.00 ^aA^	0.20 ± 0.00 ^aA^	0.20 ± 0.00 ^aA^
	2	0.20 ± 0.00 ^aA^	0.10 ± 0.00 ^aA^	0.20 ± 0.00 ^aA^	0.20 ± 0.00 ^aA^	0.20 ± 0.00 ^aA^
n-3 (%)	0	0.60 ± 0.00 ^aA^	0.90 ± 0.00 ^aA^	0.60 ± 0.00 ^aA^	0.60 ± 0.00 ^aA^	0.70 ± 0.00 ^aA^
	2	0.70 ± 0.00 ^aA^	0.90 ± 0.00 ^aA^	0.60 ± 0.00 ^aA^	0.60 ± 0.00 ^aA^	0.70 ± 0.00 ^aA^
n-6 (%)	0	9.40 ± 0.14 ^aA^	15.50 ± 0.14 ^aC^	9.85 ± 0.07 ^aA^	9.15 ± 0.21 ^aA^	10.80 ± 0.57 ^bB^
	2	9.70 ± 0.14 ^bA^	15.90 ± 0.14 ^bC^	9.80 ± 0.14 ^aA^	9.30 ± 0.00 ^bA^	10.30 ± 0.00 ^aB^
Cholesterol (mg/100 g of product)	0	101.80 ± 1.84 ^aB^	117.90 ± 5.23 ^aC^	100.90 ± 2.26 ^aA^	98.60 ± 1.41 ^aA^	99.40 ± 0.99 ^bA^
	2	99.70 ± 3.39 ^aB^	119.15 ± 9.40 ^aC^	96.85 ± 3.75 ^aB^	98.45 ± 6.15 ^aB^	87.00 ± 2.69 ^aA^

C—control treatment with the addition of salt, P—control treatment with the addition of sodium nitrite, SCH1—research treatment with the addition of salt and *L. plantarum* SCH1; BAL6—research treatment with the addition of salt and *P. pentosaceus* BAL6; KL14—research treatment with the addition of salt and *P. pentosaceus* KL14; SFA—total saturated fatty acids; MUFA—total monounsaturated fatty acids; PUFA—total polyunsaturated fatty acids; trans—total trans fatty acids; n-3—total fatty acids n-3; n-6—total fatty acids n-6. ^A–D^ Means in the same row followed by different uppercase letters between the treatments are significantly different (*p* < 0.05). ^a,b^ Means in the same column followed by different lowercase letters between the time are significantly different (*p* < 0.05).

**Table 2:** pH value, water activity (a_w_), oxidation-reduction potential, and TBARS index of the tested sausages.

Parameter	Time (Month)	Treatment				
		C	P	SCH1	BAL6	KL14
pH	0	5.41 ± 0.07 ^aA^	5.65 ± 0.02 ^aC^	5.50 ± 0.01 ^aB^	5.56 ± 0.01 ^aB^	5.51 ± 0.01 ^aB^
	1	5.72 ± 0.03 ^bB^	5.88 ± 0.02 ^bC^	5.62 ± 0.02 ^bA^	5.77 ± 0.03 ^bB^	5.80 ± 0.02 ^bB^
	2	5.67 ± 0.06 ^bB^	5.85 ± 0.00 ^bB^	5.50 ± 0.02 ^aA^	5.50 ± 0.02 ^aA^	5.58 ± 0.01 ^aA^
a_w_	0	0.86 ± 0.01 ^aA^	0.83 ± 0.01 ^cA^	0.87 ± 0.01 ^cA^	0.87 ± 0.01 ^bA^	0.89 ± 0.01 ^cA^
	1	0.87 ± 0.01 ^aB^	0.71 ± 0.01 ^bA^	0.79 ± 0.02 ^bA^	0.82 ± 0.00 ^bA^	0.80 ± 0.01 ^bA^
	2	0.87 ± 0.03 ^aB^	0.65 ± 0.04 ^aA^	0.68 ± 0.04 ^aA^	0.69 ± 0.01 ^aA^	0.73 ± 0.04 ^aA^
ORP (mV)	0	334.93 ± 2.91 ^aA^	320.37 ± 1.45 ^aA^	366.13 ± 11.78 ^bB^	354.43 ± 3.86 ^bB^	332.10 ± 5.57 ^aA^
	1	328.73 ± 1.14 ^aA^	312.40 ± 2.31 ^aA^	335.80 ± 4.65 ^aB^	336.23 ± 8.52 ^aB^	321.33 ± 2.44 ^aA^
	2	392.30 ± 5.27 ^bB^	386.40 ± 2.72 ^bB^	386.30 ± 2.21 ^cB^	367.80 ± 2.43 ^bA^	377.87 ± 3.60 ^bA^
TBARS (mg MDA/kg of product)	0	0.45 ± 0.06 ^aA^	0.48 ± 0.03 ^aA^	1.53 ± 0.06 ^aB^	0.45 ± 0.07 ^aA^	0.41 ± 0.01 ^aA^
	1	0.35 ± 0.04 ^aA^	0.45 ± 0.06 ^aA^	1.26 ± 0.18 ^aB^	0.58 ± 0.04 ^aA^	0.39 ± 0.03 ^aA^
	2	0.49 ± 0.01 ^aA^	0.55 ± 0.06 ^aA^	2.46 ± 0.21 ^bB^	0.39 ± 0.01 ^aA^	0.36 ± 0.04 ^aA^

C—control treatment with salt, P—control treatment with salt nitrite, SCH1—treatment with *L. plantarum* SCH1; BAL6—treatment with *P. pentosaceus* BAL6; KL14—treatment with *P. pentosaceus* KL14. The values are expressed as means ± standard deviation. ^A–C^ Means in the same row followed by different uppercase letters between the treatments are significantly different (*p* < 0.05). ^a–c^ Means in the same column followed by different lowercase letters between the time are significantly different (*p* < 0.05).

**Table 3:** Color measurement of the tested sausages.

Parameter	Time (Month)	Treatment				
		C	P	SCH1	BAL6	KL14
L*	0	49.15 ± 2.86 ^bB^	45.70 ± 2.87 ^bA^	52.33 ± 4.62 ^aB^	49.95 ± 5.69 ^aB^	52.20 ± 4.19 ^aB^
	1	49.08 ± 2.66 ^bB^	42.82 ± 2.47 ^aA^	52.19 ± 3.39 ^aB^	51.34 ± 2.85 ^aB^	50.18 ± 4.13 ^aB^
	2	46.08 ± 2.68 ^aA^	46.64 ± 2.34 ^bA^	54.92 ± 3.71 ^aB^	55.59 ± 2.91 ^bB^	53.87 ± 3.75 ^bB^
a*	0	7.16 ± 2.38 ^aB^	9.37 ± 0.78 ^cC^	5.25 ± 2.14 ^bB^	4.98 ± 2.72 ^aA^	9.10 ± 2.63 ^aB^
	1	8.69 ± 1.19 ^bD^	5.80 ± 2.23 ^aC^	3.43 ± 2.67 ^aA^	6.85 ± 1.14 ^bB^	8.74 ± 1.90 ^aD^
	2	9.29 ± 1.52 ^bC^	7.23 ± 1.99 ^bB^	3.58 ± 2.28 ^aA^	6.80 ± 1.74 ^bB^	8.31 ± 1.52 ^aB^
b*	0	6.88 ± 2.65 ^aA^	7.24 ± 0.86 ^bA^	6.95 ± 1.45 ^aA^	6.76 ± 1.64 ^aA^	5.84 ± 1.37 ^aA^
	1	5.81 ± 1.19 ^aA^	5.79 ± 0.84 ^aA^	8.03 ± 2.44 ^bB^	6.55 ± 1.06 ^aA^	5.53 ± 1.00 ^aA^
	2	7.27 ± 1.12 ^bA^	8.02 ± 0.97 ^cB^	9.18 ± 1.53 ^bB^	6.93 ± 0.90 ^aA^	6.80 ± 0.84 ^bA^

L*a*b*—color parameters, C—control treatment with salt, P—control treatment with salt nitrite, SCH1—treatment with *L. plantarum* SCH1; BAL6—treatment with *P. pentosaceus* BAL6; KL14—treatment with *P. pentosaceus* KL14. The values are expressed as means ± standard deviation. ^A–D^ Means in the same row followed by different uppercase letters between the treatments are significantly different (*p* < 0.05). ^a–c^ Means in the same column followed by different lowercase letters between the times are significantly different (*p* < 0.05).

**Table 4:** Microbial assessment of the tested sausages.

Parameter	Time (Month)	Treatment				
		C	P	SCH1	BAL6	KL14
TVC (log CFU/g)	0	9.55 ± 0.26 ^aB^	9.36 ± 0.33 ^aB^	9.13 ± 0.08 ^aA^	9.86 ± 0.38 ^aB^	9.79 ± 0.06 ^aB^
	1	10.48 ± 0.04 ^bA^	10.56 ± 0.08 ^bA^	10.47 ± 0.49 ^bA^	10.29 ± 0.30 ^bA^	10.27 ± 0.51 ^bA^
	2	10.65 ± 0.22 ^bA^	10.73 ± 0.02 ^bA^	10.63 ± 0.28 ^bA^	10.63 ± 0.02 ^cA^	10.78 ± 0.01 ^cA^
LAB (log CFU/g)	0	6.52 ± 0.25 ^bA^	7.74 ± 0.37 ^cC^	7.09 ± 0.12 ^cB^	8.50 ± 0.71 ^bD^	7.92 ± 0.11 ^bC^
	1	6.15 ± 0.21 ^aA^	6.22 ± 0.25 ^aA^	6.21 ± 0.13 ^aA^	9.29 ± 0.01 ^cB^	8.26 ± 0.06 ^cB^
	2	6.22 ± 0.11 ^aA^	6.71 ± 0.35 ^bA^	6.42 ± 0.11 ^bA^	7.31 ± 0.01 ^aB^	7.70 ± 0.01 ^aB^
ENT (log CFU/g)	0	<1.00	<1.00	<1.00	<1.00	<1.00
	1	<1.00	<1.00	<1.00	<1.00	<1.00
	2	<1.00	<1.00	<1.00	<1.00	<1.00
EC (log CFU/g)	0	<1.00	<1.00	<1.00	<1.00	<1.00
	1	<1.00	<1.00	<1.00	<1.00	<1.00
	2	<1.00	<1.00	<1.00	<1.00	<1.00
ST (log CFU/g)	0	<1.00	<1.00	<1.00	<1.00	<1.00
	1	<1.00	<1.00	<1.00	<1.00	<1.00
	2	<1.00	<1.00	<1.00	<1.00	<1.00
YM (log CFU/g)	0	5.19 ± 0.04 ^aB^	3.10 ± 0.12 ^aA^	4.15 ± 0.15 ^aB^	2.38 ± 0.04 ^aA^	4.60 ± 0.20 ^aB^
	1	5.23 ± 0.06 ^aB^	3.45 ± 0.08 ^aA^	4.38 ± 0.04 ^aB^	2.78 ± 0.10 ^aA^	4.64 ± 0.06 ^aB^
	2	5.66 ± 0.14 ^aB^	3.00 ± 0.00 ^aA^	4.38 ± 0.18 ^aB^	2.80 ± 0.02 ^aA^	4.80 ± 0.10 ^aB^
SAL	0	nd	nd	nd	nd	nd
	1	nd	nd	nd	nd	nd
	2	nd	nd	nd	nd	nd
CAMP	0	nd	nd	nd	nd	nd
	1	nd	nd	nd	nd	nd
	2	nd	nd	nd	nd	nd

C—control treatment with salt, P—control treatment with salt nitrite, SCH1—treatment with *L. plantarum* SCH1; BAL6—treatment with *P. pentosaceus* BAL6; KL14—treatment with *P. pentosaceus* KL14; TVC—total viable count, LAB—lactic acid bacteria, ENT—bacteria from *Enterobacteriaceae* family, EC—*Escherichia coli*, ST—number of coagulase-positive staphylococci (*Staphylococcus aureus* and other species), YM—yeasts and molds; SAL—*Salmonella* spp., CAMP—*Campylobacter* spp.; <1.00—below the level of detection; nd—not detected. The values are expressed as means ± standard deviation. ^A–D^ Means in the same row followed by different uppercase letters between the variants are significantly different (*p* < 0.05). ^a–c^ Means in the same column followed by different lowercase letters between the times are significantly different (*p* < 0.05).

## Data Availability

All of the data is contained within the article.
